# Shut Down, Don't Stress Out

**DOI:** 10.1371/journal.pbio.0020373

**Published:** 2004-10-05

Among the many stresses faced by a cell, one of the most serious is exposure to oxidizing agents. An invading organism, for example, must defend itself against the oxidative assault mounted by a host's immune system. Since oxidation can rapidly destroy many types of molecules, cells have developed multiple means of protecting against it. Rapid mobilization of these defenses requires diversion of resources and temporary suspension of many normal cellular functions, including protein synthesis. In a new study, Elise Hondorp and Rowena Matthews show that when the Escherichia coli bacterium confronts oxidative stress, an enzyme that stands at a central point in the amino acid supply line for protein synthesis is rapidly and reversibly inactivated.

Of the twenty amino acids that make up proteins, methionine plays a special role. It is the first amino acid added to every polypeptide chain, and without it, protein synthesis grinds quickly to a halt. Methionine is formed in E. coli through the action of the enzyme cobalamin-independent methionine synthase (MetE), which makes up between one and five percent of all protein in the cell. Thus, by turning off MetE in the face of oxidative stress, protein synthesis can be slowed or stopped, freeing cellular resources to be used elsewhere.

Hondorp and Matthews show that in E. coli, MetE is acutely vulnerable to oxidation under a variety of conditions. These results are in accord with a companion study by Leichert and Jakob, also in *PLoS Biology,* showing that MetE is one of the proteins most sensitive to oxidative damage. When the active site of MetE is stressed by an oxidant, Hondorp and Matthews show, it is temporarily blocked by the attachment of a glutathione subunit. Glutathione is a small molecule that includes a reactive sulfur atom. During “glutathionylation” of MetE, a sulfur on an amino acid of the enzyme is oxidized and links up with a sulfur on glutathione. This study shows that glutathionylation occurs only on a specific amino acid (cysteine 645), which recent structural work indicates sits at the entrance to the active site.

Attachment of the bulky glutathione subunit to this cysteine would be expected to block the active site, thus shutting down enzymatic activity. The results indicate that glutathionylation does indeed prevent activity of the enzyme, and furthermore, causes the enzyme to change its three-dimensional form. As the oxidative challenge abates, glutathionylation may be reversed, and the normal activity of the enzyme restored. Thus, glutathionylation of MetE may also serve to protect the active site from permanent oxidative damage. While glutathionylation is a common strategy in eukaryotes, MetE is so far one of the few proteins in bacteria known to be affected in this way.

Shutting down MetE and limiting methionine production may play another important role, namely, communicating the bacterium's metabolic state to other nearby E. coli. Methionine is a precursor for the signaling molecule AI-2, which is released extracellularly and appears to serve as a key indicator of colony health and density. This information enables neighboring cells to better respond to changing and potentially hostile environments. Thus, the glutathionylation and inactivation of MetE may provide a simple mechanism by which a bacterium and its neighbors attempt to deal with oxidative stress.

